# Fear of Cancer Recurrence and Coping Strategies among Prostate Cancer Survivors: A Qualitative Study

**DOI:** 10.3390/curroncol30070493

**Published:** 2023-07-16

**Authors:** Abbas Mardani, Mansoureh Ashghali Farahani, Alice Khachian, Mojtaba Vaismoradi

**Affiliations:** 1 Nursing and Midwifery Care Research Center, School of Nursing and Midwifery, Iran University of Medical Sciences, Tehran 1449614535, Iran or mardani.a@iums.ac.ir (A.M.); farahani.ma@iums.ac.ir (M.A.F.); 2 Faculty of Nursing and Health Sciences, Nord University, 8049 Bodø, Norway; mojtaba.vaismoradi@nord.no; 3 Faculty of Science and Health, Charles Sturt University, Orange, NSW 2800, Australia

**Keywords:** coping, continuous care, fear of cancer recurrence, prostate cancer survivors, qualitative research

## Abstract

Background: Fear of cancer recurrence (FCR), as a commonly reported problem among prostate cancer survivors, has not been fully understood. This study aimed to explore the experience of FCR and relevant coping strategies among Iranian prostate cancer survivors. Methods: Qualitative research was conducted on 13 men who completed treatments for prostate cancer in the last 24 months. The participants were selected through purposeful sampling, and in-depth semi-structured interviews were used for data collection. Conventional content analysis was used for data analysis. Results: Data analysis led to the emergence of three themes. “Living with insecurity” describes the participants’ experiences regarding what triggers FCR with two categories, including “fear of incomplete cure” and “fear of cancer return.” In addition, “struggling to cope” with two categories, including “psychological strategies” and “spiritual coping,” presents coping strategies used by the participants for reducing FCR. Furthermore, “trying to prevent cancer recurrence” with two categories, “seeking health” and “lifestyle modification,” indicates coping strategies used by the participants to prevent cancer recurrence. Conclusions: Healthcare providers need to consider the cultural characteristics of prostate cancer survivors when assessing their FCR, encourage them to disclose their concerns and fears, and provide tailored interventions in order to reduce FCR among them.

## 1. Introduction

Prostate cancer (PCa) is the second most common cancer among men after lung cancer. It consisted of 14.1% of new cases of cancer in men in 2020, and was the fifth cause of death from cancer globally for men [1]. In Iran, PCa is the second most common cancer in men and the eighth cause of death from all cancers [2]. Fortunately, early diagnosis and advances in cancer treatment have led to an unprecedented increase in the number of people who continue to live after cancer [3]. Currently, the five-year survival rate of localized PCa in developed countries is reported to be about 98% [4]. 

After finishing the treatment, PCa survivors face many challenges that affect their quality of life [5,6]. Fear of cancer recurrence (FCR) is a commonly reported problem among PCa survivors despite high survival rates [7,8,9,10]. FCR has been defined by Lebel et al. [11] as: “Fear, worry, or concern about cancer returning or progressing.” Van de Wal’s study found that 36% of PCa survivors experienced a high rate of FCR [12]. Research studies have revealed that FCR tends to rise during the post-treatment period and follow-up appointments [13,14]. A recent systematic review showed that PCa patients commonly experience symptoms related to FCR and prostate-specific antigen (PSA), such as anxiety, which significantly impact their mental health and overall quality of life [14]. A study in the USA found that those men who had positive surgical margins following radical prostatectomy (RP) experienced an increase in FCR, which was higher than men with negative surgical margins [15]. For PCa survivors, FCR is a crucial factor affecting their physical and mental health, but unfortunately, it is often disregarded during their care [14]. 

The experience and expression of emotions within a cultural context are shaped by the culture itself. FCR during cancer trajectory beyond the biological aspect is also impacted by social and environmental factors [16]. Therefore, the experiences of PCa survivors should be investigated from cultural aspects to improve patients’ experiences in this trajectory. 

Previous studies have mainly approached FCR in PCa survivors using a quantitative research approach and have investigated predictors and variables correlated with FCR [7,8,10,12,14,15,17]. There is no qualitative study that has specifically investigated FCR in PCa survivors. The rationale for focusing on FCR in PCa survivors in this study lies in the high curability rate of PCa in the early-stage disease and a five-year relative survival rate of nearly 100% [1]. In addition, based on clinical observations, it is believed that certain factors specific to PCa may play a role in the FCR. The routine monitoring of PSA levels has both clinical and psychological significance for survivors. The uncertainty surrounding an increase in PSA levels may potentially trigger FCR [14]. Accordingly, FCR in PCa survivors is expected to be different compared to other cancer types, which may have a higher mortality rate or a more aggressive disease course. 

A deep understanding of FCR and relevant coping strategies used by PCa survivors is required to find their impacts on the life structure, psychological wellbeing, and family relationships, as well as to find how optimal care can be provided to them. Therefore, the aim of this study was to explore the experience of Iranian PCa survivors regarding FCR and relevant coping strategies using a qualitative research method.

## 2. Materials and Methods

### 2.1. Design and Participants

The research method used in this study was qualitative with a conventional content analysis approach. By condensing and abstracting textual data, qualitative content analysis helps explore new perspectives and experiences on a social phenomenon [18]. The authors, in reporting this study, followed the standards for reporting qualitative research (SRQR) guidelines (Supplementary file S1) [19]. 

This study recruited participants based on specific criteria, including having completed PCa treatments within the past 24 months, having no metastasis to other organs, and being aware of the PCa diagnosis. The participants were selected through purposive sampling from July 2022 to March 2023.

### 2.2. Ethics Considerations

The Ethics Committee affiliated with the Iran University of Medical Sciences granted ethical approval for this study (decree number: IR.IUMS.REC.1401.160). Additionally, healthcare authorities permitted entry into the hospitals and also access to patients’ information. The participants were provided with comprehensive explanations about this study’s objective, voluntary participation, anonymity, the right to withdraw from the study at any point, and the confidentiality of collected data. Written informed consent and permission to audio-record interviews were obtained from all participants before data collection.

### 2.3. Data Collection

The first researcher (AM) carefully examined the medical records of PCa patients who had completed treatments within the past 24 months at four referral hospitals in an urban area of Iran to identify eligible participants. After reviewing 125 files, 68 individuals initially met the criteria, and then they were contacted to determine additional eligibility. Those who fully met the inclusion criteria were invited to participate in this study. Data collection continued until data saturation was achieved. Finally, 14 interviews were conducted with 13 PCa survivors.

The researcher (AM) conducted in-depth semi-structured face-to-face interviews in Farsi, using open-ended questions. He scheduled the interviews at a time and place that was convenient for the participants so as not to disrupt their daily life routines. The interview guide contained questions to collect comprehensive data about the research phenomenon as follows: (1) What do you think about what will happen in the future about your cancer? (2) Are you worried that your prostate cancer might come back? (3) What did you do to deal with the worries caused by the cancer coming back? (4) Did you make any life changes to prevent the cancer from coming back? The research team developed these questions after reviewing the literature on FCR and related issues among cancer survivors. To enhance the depth of the interviews, the researcher asked probing questions to explore the participants’ perspectives further.

The interviews lasted between 40 and 60 min, with 12 participants interviewed once and one participant interviewed twice to clarify ambiguities in the data collection process. To ensure the accuracy of the data, a digital audio recorder was used to record all interviews.

Before conducting the interviews, the participants were asked questions pertaining to their socio-demographic characteristics, such as age, married status, education level, employment, and economic status, cancer treatment modalities, and the time elapsed since PCa treatments.

This study was conducted by three faculty members from nursing and midwifery schools and one Ph.D. candidate who was experienced in qualitative research and had previous experiences in cancer research and care [6,20,21,22]. The interviewer (AM) had vast clinical work experiences with cancer patients, but he had no prior relationship with the participants. To avoid subjectivity, he wrote reflective notes to acknowledge his assumptions regarding the study phenomenon.

### 2.4. Data Analysis

The first author (AM) transcribed the interviews and received support from the research team (MFA, AK, MV) for data analysis concurrently with the data collection process using the conventional qualitative content analysis method [23,24]. They thoroughly read through the transcriptions multiple times to fully immerse themselves in the content and identify meaningful units within the sentences. Through open coding, they labeled the meaning units and grouped similar codes into subcategories, which were then compared to develop categories and themes in relation to the entire data set [18].

### 2.5. Trustworthiness of Data

To ensure the trustworthiness of the qualitative research, this study followed the rigor components as credibility, transferability, dependability, and confirmability. The credibility was strengthened through prolonged engagement with the participants and settings, peer debriefing, reflexivity, and member checking. The interviewer also wrote reflective notes to avoid personal subjectivity. Peer debriefing involved a third researcher reviewing the data analysis process, while member checking involved sharing research results with two participants to confirm their perspectives. To ensure transferability, a thick description of the research findings was provided. For dependability and confirmability, an audit trail was conducted by an impartial expert in qualitative research who assessed the transcripts, data analyses, and findings [25].

## 3. Results

The age of the participants varied from 48 to 73 years old. All of them were married and had different educational levels, from illiterate to academic. In addition, six of the participants were employed, and the rest were retired. The economic status of the participants ranged from sufficient to not sufficient. The majority of them (*n* = 12) had under radical prostatectomy (RP) for PCa. Furthermore, the time passed from cancer treatment varied from 2 to 24 months. Socio-demographic demographic data has been presented in Table 1. 

**Figure 1:**
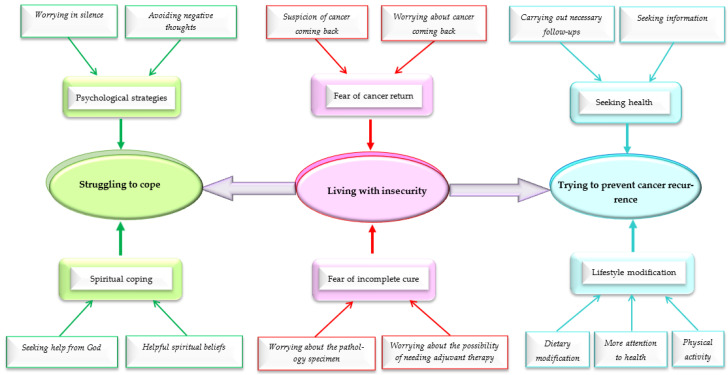
The schematic presentation of the data analysis products.

Data analysis led to 13 subcategories, 6 categories, and 3 themes showing the FCR and relevant coping strategies among Iranian men with PCa. The findings are provided in Table 2 and Figure 1.

### 3.1. Living with Insecurity

Living with insecurity describes the participants’ experiences regarding the triggers of FCR. They experienced living with insecurity about PCa status due to the fear of an incomplete cure immediately after treatment and the fear of cancer returning over time.

#### 3.1.1. Fear of Incomplete Cure

The experiences of the participants regarding the fear of incomplete cure were presented in two subcategories, including “worrying about the pathology specimen” and “worrying about the possibility of needing adjuvant therapy.”

(1) Worrying about the pathology specimen.

During RP surgery, the pathology specimen was taken from the surgical site to evaluate the surgical margins and the success of surgery in removing cancer cells. The participants, when waiting to receive the report of the pathology lasting between 15 and 20 days, had different levels of worry and stress. They did not know what was going to happen in the future according to the pathology specimen results.

“Ever since the doctor talked to me about the pathology specimen, I was worried about the answer and I was thinking about what was going to happen.”

(Participant 4)

Even the worry and stress of three of the participants were so much that they requested their family members to hand over the result of the pathology specimen to the doctor.

“When we wanted to go to the doctor’s office to show the pathology result, I did not look at the result, I mean, I did not dare to look at it at all, I asked my wife to show it to the doctor.”

(Participant 2)

One of the participants who faced an unfavorable pathology answer described his feelings as follows:

“When the doctor saw the pathology result, he said that there are still cancer cells. When I heard this, I felt that my death was near and that my cancer would not be cured.”

(Participant 13)

(2) Worrying about the possibility of needing adjuvant therapy.

The possibility of needing adjuvant therapy such as radiotherapy or chemotherapy beyond primary treatment was another source of worry in the participants leading to the fear of incomplete cure and FCR. One of the main reasons was unfavorable experiences by a family member or acquaintance who was treated using chemotherapy or radiotherapy.

“My own father had stomach cancer and he received radiotherapy and chemotherapy…he became weak little by little and then passed away after a year…when I heard that chemotherapy or radiotherapy should be done If the pathology specimen result be unfavorable… I was worried that my situation would be like him.”

(Participant 9)

Another reason related to being worried about the possibility of needing adjuvant therapy was worrying about the entanglement of family members in the process of treating and harassing them, that frequently mentioned by the participants.

“Chemotherapy or radiation therapy itself is one side of the issue, and the fact that it implicates the whole family and causes the family to suffer is another side of the issue.”.

(Participant 12)

#### 3.1.2. Fear of Cancer Return

The participants’ experiences regarding the fear of cancer return were placed into two subcategories, including “worrying about cancer coming back” and “suspicion of cancer coming back.”

(1) Worrying about cancer coming back.

The participants had worries about the recurrence of their cancer and the changing and deterioration of their life situation. The most important situations that increased their worries about cancer coming back were related to the time of checking PSA test, awaiting to receive the result, and follow-up appointments. These issues were especially more severe when the participants checked the PSA for the first time and were waiting to inform of the result to the doctor in follow-up appointments.

“When I went to perform this test [PSA] and even close to the time of the test, I was very worried about what the result of this test would be… this worry between taking this test and awaiting to get the result and show it to the doctor became much more and I had stress.”

(Participant 4)

In addition, the Gleason’s score and International Society of Urological Pathology (ISUP) grade was an influential factor in worrying the participants about cancer’s recurrence. Those participants whose doctor had given them information about a high Gleason score at diagnosis were more concerned about it.

“The doctor said that my cancer score was 8 and ‘if you had come a little later, it was not clear what would happen to you.’ The fact that my cancer is advanced at the time of diagnosis makes me more worried about that my cancer might come back one day.”

(Participant 8)

An important cause for making the participants worried was their concerns about the situation of their family in case of cancer recurrence and metastasis to other organs.

“I always think that if my cancer returns what will happen to me, my children are girls; I think about these things.”

(Participant 9)

(2) Suspicion of cancer coming back.

Some symptoms and events ignited the participants’ suspicion of the recurrence of cancer. They were mostly urinary symptoms such as swelling and pain in the testicles, the presence of blood in the urine, difficulty in urinating, and frequent occurrences of urinary obstruction.

“About one month after that I was discharged from the hospital, I do not know what was the problem that caused my urination to be drop by drop. During that month, I was very bothered when urinating, which made me suspect that my cancer has relapsed.”

(Participant 8)

Two participants were suspected of cancer metastasis to other organs due to severe back pain and pelvic bone pain.

“It was 10 months after my surgery that I had severe back pain. I had already read on the Internet about the recurrence of prostate cancer and its metastasis, and I knew that prostate cancer might give metastasis to bones and vertebrae of my back. I thought that cancer had spread in my body.”

(Participant 11)

### 3.2. Struggling to Cope

It describes the participants’ experiences with the use of coping strategies to overcome their worries and fears related to insecurity caused by the fear of incomplete cure and cancer recurrence. Two main strategies, including psychological strategies and spiritual coping, to modify their worries and fears were used.

#### 3.2.1. Psychological Strategies

The participants used two main psychological strategies, including “worrying in silence” and “avoiding negative thoughts” to cope with insecurity raised by an incomplete cure and fear of cancer recurrence.

(1) Worrying in silence.

The participants tried to keep their concerns to themselves and not express them. In many situations, despite being worried about the result of the pathology specimen, the PSA test, and the recurrence of their cancer, they tried to appear good and calm. They often hide their worries to avoid worrying about their wife and children.

“I did not want my wife and children to know my concern about the possibility of cancer recurrence and they would also become worried.”

(Participant 10)

(2) Avoiding negative thoughts.

Another psychological strategy used by some participants was to avoid negative thoughts. They tried not to think about the recurrence of cancer at all. Even one of them believed that if you think about the recurrence of cancer, your cancer will eventually recur.

“I try not to think about it at all; there is a law called the law of attraction, and in my opinion, whatever you think about, you attract it… if I think about cancer returning, I think this issue will happen. That is why I do not think about it at all.”

(Participant 13)

Some participants tried not to think about the recurrence of cancer or incomplete cure by making themselves busy with other things.

“When the thought of cancer recurrence comes to me, I try not to give too much attention to it… At such times, I make myself busy with other things, for example, I go to watch the TV or read a book.”

(Participant 12)

#### 3.2.2. Spiritual Coping

Two spiritual coping strategies, including “seeking help from God” and “helpful spiritual beliefs” helped the participants to cope with the fear of incomplete cure and cancer recurrence.

(1) Seeking help from God.

The participants mostly sought help from God in dealing with the worries of cancer recurrence by praying and trusting in God. One of them referred that talking to God in times of worry was very calming.

“When I was waiting for the PSA test result, I was very stressed, I talked to God and asked for his help, and this was relaxing for me down.”

(Participant 1)

And another said about her talk with God:

“I ask God to take care of me and I still have a lot of work to do and I have three daughters that I have to take care of, just talking to God helps me a lot to deal with this worry.”

(Participant 9)

(2) Helpful spiritual beliefs.

Some spiritual beliefs used by the participants helped them to cope with their worries regarding the fear of an incomplete cure and cancer recurrence. Some of them talked about the certainty of human death and believed that since all human beings will die one day and the time of their death is determined by God, they do not see the need to be worried too much about the recurrence or metastasis of cancer to other organs.

“The world is a place of passage, and this world is a passage and not for permanent residency; after all, we will all leave this world one day and I have to leave one day. Therefore, I am not too worried about cancer recurrence.”

(Participant 6)

Acceptance of divine predestination was another belief stated by the participants, which acted as a factor in coping with worries caused by cancer recurrence.

“I always tell God that I will accept whatever you decide for me and I have no complaints against you. I believe that God wanted to give me this cancer and nothing will happen until God wills and this belief helps me to better deal with this worry of recurrence.”

(Participant 4)

Even some participants stated that they entrusted themselves and their destiny to God and in this way, they coped with their worry about the possibility of needing adjuvant therapy and cancer recurrence.

“I left everything to God and I think this is the best thing that will reduce my worries about cancer recurrence.” .

(Participant 5)

### 3.3. Trying to Prevent Cancer Recurrence

This theme describes strategies used by the participants as their efforts and responsibility to prevent cancer recurrence. They modified their lifestyles where they felt they could be useful and sought health support. 

#### 3.3.1. Seeking Health

The participants, through “seeking information” and “carrying out necessary follow-ups” tried to promote their health and prevent the recurrence of cancer.

(1) Seeking information.

Seeking information was one of the strategies used by the participants to acquire the required information regarding how to prevent cancer recurrence. They sought this information from various sources such as friends who were health care providers and from the Internet.

“I read a lot on the internet about the things that should be done to prevent cancer recurrence, and I tried to incorporate them into my life if they made sense.”

(Participant 11)

However, information on how to cope with FCR and prevent cancer recurrence was not provided to the participants by healthcare providers.

“One of the issues I expected to get information about was the issue of cancer relapse and what I need to do to prevent it from coming back, but no one talked to me about that.”

(Participant 13)

(2) Carrying out necessary follow-ups.

To prevent the recurrence of cancer, the participants performed necessary follow-ups according to the recommendations made by the healthcare provider’s team. They had regular follow-up appointments and performed checking PSA levels as recommended and would inform their doctor about the result.

“The doctor told me that this test (PSA) could show the state of my cancer and I performed this test based on the doctor’s advice.”

(Participant 1)

The participants who suspected their cancer recurrence informed their doctor as soon as possible.

“Pain in my pelvic bones occurred about 5 to 6 months after the end of radiotherapy. I thought that my cancer had metastasized to my pelvic bones, so I immediately informed my doctor.”

(Participant 13)

#### 3.3.2. Lifestyle Modification

The participants took responsibility and made changes in their lifestyle in three aspects, including “physical activity”, “more attention to health”, and “dietary modification” in order to prevent cancer recurrence.

(1) Physical activity.

Physical activity usually was performed in the form of a regular daily walking program by most of the participants. However, one of the participants chose a regular pool program to increase his physical activity and prevent cancer recurrence. Some of the participants even decided not to use walking instead of using vehicles to perform their daily tasks to remain physically active.

“I know that regular physical activity can reduce the risk of cancer recurrence, so I walk every day. Also, sometimes when I have something to do, I try not to use my car or taxi and go on foot.”

(Participant 9)

(2) More attention to health.

They paid more attention to their health status after cancer treatment to prevent cancer recurrence and deterioration of their health status. They knew that stress was an important factor in cancer recurrence and tried to avoid stress. Although avoiding stress has little effect on preventing cancer recurrence based on scientific evidence, cultural beliefs can shape the practice of the participants [26].

“I do not stress myself for trivial issues anymore, because I know that stress is the cause of many diseases and may even cause my cancer to come back.”

(Participant 11)

In some cases, they started to modify their bad habits to prevent cancer recurrence, for example, stopping smoking and eating meals at the right time.

“Perhaps the most important change that I made in my life after cancer was related to smoking. I had been smoking for more than 30 years. When I was in the hospital, I asked the doctor if smoking would have an effect on my cancer. He said that it is better not to smoke and that it may affect the return of your cancer or even other cancers, so after the surgery, I reduced the amount of smoking and I stopped smoking completely three months after the surgery.”

(Participant 9)

(3) Dietary modification.

It was an important strategy to reduce the risk of cancer recurrence. It included reducing food consumption, avoiding harmful foods such as fast foods, reducing salt, sugar, and fat consumption, using organic food, and incorporating fruits and vegetables into the daily diet.

“I include fruit three times a day in my diet, one in the morning, one in the evening, and one at night, or for example, I read somewhere that some types of tea have additives that may be related to cancer, and after that, I only use organic tea without additives.”

(Participant 12)

## 4. Discussion

This study yielded specific insights about FCR in PCa survivors. Furthermore, this is the pioneering research to investigate FCR in Iranian men with PCa. Our findings highlighted the nature of FCR among the PCa survivors and indicated what strategies were used by them to manage FCR.

The participants experienced insecurity in their life due to the fear of an incomplete cure and the fear of cancer return. Fear of an incomplete cure was triggered by worrying about the pathology specimen and the possibility of needing adjuvant therapy. Previous research also has shown the experience of the survivors of various cancers regarding their concerns and doubts about the results of treatment and incomplete treatment. Similarly, a phenomenological study in Iran on the survivors of multitype of cancer reported that one of the most important concerns of the survivors was about the effectiveness of the treatment [27]. Another qualitative study in Canada found that one of the most important concerns of women with breast cancer was the complete eradication of the cancer [28]. Furthermore, one of the important experiences of Chinese women with breast cancer prior to adjuvant chemotherapy was the complex psychological pressure associated with chemotherapy and their family [29], which is similar to our findings regarding being worried about the possibility of needing adjuvant therapy. Also, previous studies showed that treatment failure and positive surgical margin were associated with high FCR among PCa survivors [9,17]. 

Worrying about cancer coming back was formed with a focus on PSA tests and follow-up appointments. The suspicion of the cancer coming back was based on the participants’ experiences of physical symptoms. A qualitative study by Vyas et al. in the United Kingdom showed that patients with PCa, after completing treatments, were still concerned about the possibility of cancer returning in the future. According to their study, follow-up appointments, repeat scans, and especially current PSA values continued to cause ongoing stress and anxiety [30], which are in line with our findings. The findings of a quantitative study in the Netherlands found that high FCR in PCa survivors was related to PSA anxiety and disease-related symptoms, and medical examinations [12]. In a qualitative study by Zhang et al. on Chinese cancer survivors, disease symptoms and awaiting medical examination were FCR triggers [31]. Therefore, it is important to monitor FCR in PCa survivors immediately after treatment to identify those who may be vulnerable and provide timely psychological support. This emphasizes the practical significance of the study’s findings. A recent systematic review demonstrated that cognitive behavioral therapies were successful in reducing FCR [32].

Although this study explored FCR in PCa survivors who underwent curative treatments for PCa, active surveillance has been deemed the gold standard for managing low-risk PCa in the last decade. We did not find qualitative studies that investigated FCR among PCa survivors who were undergoing active surveillance. It may be imagined lower levels of FCR in survivors undergoing active surveillance due to their lack of treatment-related adverse effects and the high cancer-specific survival rates associated with low-risk PCa. However, the findings of quantitative studies that investigated FCR in this group are contradictory. A study in Norway found that PCa survivors who underwent active surveillance had a higher FCR compared to those who underwent RP. In the case of active surveillance patients, younger age was linked to a higher FCR, while in RP patients, high PSA at diagnosis, biochemical recurrence, positive surgical margin, and fatigue were all associated with a higher FCR [17]. In addition, Irish active surveillance patients had higher FCR when compared to their RP counterparts [33]. On the contrary, some studies have shown lower levels of FCR and anxiety among PCa survivors undergoing active surveillance [34,35]. The severity of FCR and the causes leading to FCR seem to vary among PCa survivors who are undergoing curative treatments and active surveillance, and further in-depth studies are still needed in this field.

Participants in this study tried to cope with FCR by applying psychological and spiritual strategies. Worrying in silence and avoiding negative thoughts were psychological strategies for coping with worries regarding FCR. These psychological strategies are considered avoiding coping strategies against FCR. To protect their family, the participants often did not express their concerns about cancer recurrence. A qualitative study in the United Kingdom showed that PCa survivors often kept worries about FCR to themselves and did not openly discuss their fears [30]. In addition, similar experiences can be found in the literature related to the fear of recurrence in cancer survivors. The findings of a qualitative study conducted among Taiwanese breast cancer survivors indicated that participants hide their sufferings about the FCR to protect their families [36]. Also, keeping FCR to oneself and keeping themselves busy were documented in Chinese cancer survivors [31], which is in line with our findings. It seems that hiding worries about the FCR among Iranian PCa survivors is also deeply influenced by cultural context. As the pillar of the family in Iran, the man has the most important role in the family and is considered the main supporter and provider of financial needs [37]. It is expected that they will remain strong even in times of illness. Also, the stigma associated with cancer, and especially PCa, in Iranian culture may prevent them from disclosing cancer and their feelings and worries about it [38,39]. Hence, it is crucial to take into account these inclinations of PCa survivors in clinical practice. There is a pressing need for healthcare institutions in Iran to plan tailored survivorship care to meet these aspects of FCR among PCa survivors. 

The participants utilized spiritual coping strategies, including seeking help from God and helpful spiritual beliefs, to manage FCR and minimize its negative impacts. Embracing faith, trust in God, and prayer resulted in a decrease in insecurity and FCR. The indirect relationship between spiritual wellbeing and coping and FCR has been documented in previous studies [40,41,42]. While this issue has not been extensively studied in Western societies, it has been observed in certain studies conducted in Eastern nations [31,40]. Spiritually-oriented coping mechanisms are crucial aspects of Iranian and Eastern beliefs and traditions.

Moreover, the participants in the present study responded to their FCR by modifying their lifestyle and seeking health in order to prevent cancer recurrence. Although the positive effects of making lifestyle changes to prevent cancer progression or recurrence in cancer survivors have become more apparent, there is limited knowledge of how these behaviors affect FCR. We only found a study that indicated a direct relationship between applying physical activity and a healthy diet and the reduction of FCR among cancer survivors [43]. Although the participants in the present study were actively performing follow-up tests and appointments, as well as seeking information to prevent cancer recurrence, they stated that they did not receive any information about the fear of recurrence or prevention of cancer recurrence. By offering survivors psychological and informational support, healthcare providers can enhance PCa survivors’ emotional wellbeing and level of psychosocial adaptation [44].

Although the current study was conducted inductively, and the findings were extracted from the stories of the participants without using a specific theoretical framework, the findings can contribute to enriching the literature and theories related to the FCR. Among researchers, the explanatory model developed by Lee-Jones is frequently referenced for FCR [45]. Lee-Jones and colleagues introduced a theoretical framework that draws from Leventhal’s Self-Regulation Model of Illness. This model suggests that both external and internal stimuli contribute to an individual’s perception of a physical ailment or health risk, along with associated emotions such as fear or distress. These perceptions then inform the individual’s coping strategies [46]. The findings of our study can be explained by this theoretical framework and contribute to its strengthening. Various internal and external stimuli, such as the fear of an incomplete cure or the fear of cancer returning and experiencing physical symptoms, trigger the FCR among PCa survivors. They respond to these stimuli by applying avoidance (worrying in silence and avoiding negative thoughts) and positive coping strategies (spiritual coping, seeking health, and lifestyle modification). 

The findings of this study should be interpreted with caution due to various limitations. The researchers tried to remain impartial through reflexivity; their subjectivity as qualified nurses with a specific interest in the topic might have influenced the interview process. Also, this study was conducted only in an urban area of Iran which might influence the transferability of the findings to other contexts. A small group of men with PCa who mostly underwent RP without metastasis was included in this study. Therefore, to validate our findings, larger studies that incorporate both qualitative and quantitative methods and involve more diverse survivor populations in terms of socio-demographics and clinical characteristics are required. Future qualitative studies to explore FCR among PCa survivors undergoing active surveillance should be performed. 

## 5. Conclusions

The present study’s findings indicate the triggers of FCR and strategies to cope with those among men with PCa. Culture’s values and beliefs play an important role in the management of FCR. Among Iranian PCa survivors, psychological and spiritual coping strategies are deeply influenced by cultural context. The participants experience worries in silence due to their role in the family and also due to the stigma attached to PCa cancer. In addition, spiritual coping strategies have a key role in modifying FCR due to the context of Eastern nations. 

Healthcare providers need to consider FCR triggers and the cultural characteristics of PCa survivors when assessing their FCR during follow-up appointments. It is important to engage in open communication with patients and provide guidance on how to confront and manage their inner fears, allowing them to express negative emotions such as anxiety, depression, and fear in a proactive manner. In addition, survivors should be encouraged to seek support and disclose concerns and fears with healthcare providers, families, and friends rather than hiding them. In addition, it is suggested that psychological support services are developed in healthcare settings to provide tailored interventions in order to reduce FCR among PCa survivors.

## Figures and Tables

**Table 1:** The participants’ socio-demographic data.

Participant	Age (Year)	Marital Status	Education Level	Occupation	Economic Status (Self-Report)	Gleason Score /ISUP ^1^ Grade	Treatment	Time Passed from Cancer Treatment (Months)
1	62	Married	Under diploma	Employed	Relatively sufficient	8/4	RT ^2^ + CT ^3^	2
2	56	Married	Academic	Retired	Relatively sufficient	4 + 3/3	RP ^4^	24
3	70	Married	Under diploma	Retired	Relatively sufficient	4 + 3/3	RP	19
4	57	Married	Diploma	Employed	Not sufficient	3 + 4/2	RP	6
5	73	Married	Diploma	Employed	Not sufficient	6/1	RP	20
6	53	Married	Under diploma	Employed	Sufficient	4 + 3/3	RP	8
7	64	Married	Academic	Retired	Sufficient	3 + 4/2	RP	22
8	73	Married	Academic	Retired	Sufficient	8/4	RP	16
9	62	Married	Diploma	Employed	Not sufficient	4 + 3/3	RP	17
10	58	Married	Illiterate	Retired	Relatively sufficient	4 + 3/3	RP	15
11	61	Married	Academic	Retired	Sufficient	3 + 4/2	RP	14
12	67	Married	Under diploma	Retired	Sufficient	4 + 3/3	RP + HT ^5^	13
13	48	Married	Academic	Employed	Not sufficient	8/4	RT + RT + HT	23

^1^ International Society of Urological Pathology; ^2^ Radiotherapy; ^3^ Chemotherapy; ^4^ Radical Prostatectomy; ^5^ Hormone Therapy.

**Table 2:** Subcategories, categories, and themes developed in this study.

Subcategory	Category	Theme
Worrying about the pathology specimen Worrying about the possibility of needing adjuvant therapy	Fear of incomplete cure	Living with insecurity
Worrying about cancer coming back Suspicion of cancer coming back	Fear of cancer return	
Worrying in silence Avoiding negative thoughts	Psychological strategies	Struggling to cope
Seeking help from God Helpful spiritual beliefs	Spiritual coping	
Seeking information Carrying out necessary follow-ups	Seeking health	Trying to prevent cancer recurrence
Physical activity More attention to health Dietary modification	Lifestyle modification	

## Data Availability

The interview transcripts will not be made publicly available due to maintaining the confidentiality of data.

## Supplementary Materials

The following supporting information can be downloaded at: https://www.mdpi.com/article/10.3390/curroncol30070493/s1, Supplementary file S1: The standards for reporting qualitative research (SRQR) guidelines.
